# Targeting the FGF/FGFR axis and its co-alteration allies

**DOI:** 10.1016/j.esmoop.2022.100647

**Published:** 2022-11-29

**Authors:** Y. Uehara, S. Ikeda, K.H. Kim, H.J. Lim, J.J. Adashek, H.E. Persha, R. Okamura, S. Lee, J.K. Sicklick, S. Kato, R. Kurzrock

**Affiliations:** 1 Department of Precision Cancer Medicine, Center for Innovative Cancer Treatment, Tokyo Medical and Dental University, Tokyo; 2 Department of Thoracic Oncology and Respiratory Medicine, Tokyo Metropolitan Cancer and Infectious Diseases Center, Komagome Hospital, Tokyo, Japan; 3 Center for Personalized Cancer Therapy, Division of Hematology/Oncology, Department of Medicine, University of California San Diego, Moores Cancer Center, La Jolla, USA; 4 Division of Hematology and Medical Oncology, Department of Internal Medicine, Seoul National University Boramae Medical Center, Seoul; 5 Department of Internal Medicine, Veterans Health Service Medical Center, Seoul, Republic of Korea; 6 Department of Oncology, The Sidney Kimmel Comprehensive Cancer Center, The Johns Hopkins Hospital, Baltimore; 7 Purdue University College of Pharmacy, Purdue University, West Lafayette, USA; 8 Department of Surgery, Kyoto University Hospital, Kyoto, Japan; 9 Center for Personalized Cancer Therapy and Division of Surgical Oncology, Department of Surgery, UC San Diego Moores Cancer Center, La Jolla, USA; 10 WIN Consortium for Personalized Cancer Therapy, Paris, France; 11 Medical College of Wisconsin Cancer Center and Genome Science and Precision Medicine Center, Milwaukee, USA; 12 University of Nebraska (adjunct), Lincoln, Nebraska

**Keywords:** precision cancer medicine, drug resistance, tyrosine kinase inhibitor, matched therapy, combination therapy, FGFR

## Abstract

**Background:**

We analyzed the *FGF/FGFR* and co-alteration cancer landscape, hypothesizing that combination therapy might be useful in the presence of co-drivers.

**Materials and methods:**

We describe *FGF/FGFR*-altered pathways, prognosis, and co-alterations [cBioPortal (*N* = 7574)] and therapeutic outcomes [University of California San Diego Molecular Tumor Board (MTB) (*N* = 16)].

**Results:**

Patients whose cancers harbored *FGF/FGFR* alterations (*N* = 1074) versus those without them (*N* = 6500) had shorter overall survival (OS) (median: 23.1 versus 26.4 months, *P* = 0.038) (cBioPortal). Only 6.1% (65/1074 patients) had no pathogenic co-alterations accompanying *FGF/FGFR* axis abnormalities. The most frequently co-altered pathways/genes involved: *TP53* (70%); cell cycle (58%); PI3K (55%); and receptor tyrosine kinases and mitogen-activated protein kinase (MAPK) (65%). Harboring alterations in both *FGF/FGFR* and in the *TP53* pathway or in the cell cycle pathway correlated with shorter OS (versus *FGF/FGFR*-altered without those co-altered signals) (*P* = 0.0001 and 0.0065). Four of 16 fibroblast growth factor receptor (FGFR) inhibitor-treated patients presented at MTB attained durable partial responses (PRs) (9, 12, 22+, and 52+ months); an additional two, stable disease (SD) of ≥6 months (13+ and 15 months) [clinical benefit rate (SD ≥ 6 months/PR) = 38%]. Importantly, six patients with cyclin pathway co-alterations received the CDK4/6 inhibitor palbociclib (75 mg p.o. 3 weeks on, 1 week off) and the multikinase FGFR inhibitor lenvatinib (10 mg p.o. daily); three (50%) achieved a PR [9 (ovarian), 12 (biliary), and 52+ months (osteosarcoma)]. Palbociclib and lenvatinib were tolerated well.

**Conclusions:**

*FGF/FGFR* alterations portend a poor prognosis and are frequently accompanied by pathogenic co-aberrations. Malignancies harboring co-alterations that activate both cyclin and FGFR pathways can be co-targeted by CDK4/6 and FGFR inhibitors.

## Highlights

• Overall, 93.9% of cancers bearing *FGF/FGFR* axis genomic alterations also harbored deleterious co-alterations.
• Co-alterations occurred in critical pathways: *TP53*, cell cycle, PI3K, tyrosine kinase, and MAPK.
• *TP53* and cell cycle co-alterations correlated with shorter survival versus *FGF/FGFR*-altered without those co-aberrations.
• Patients (3/6) with *FGF/FGFR* and cyclin alterations responded to lenvatinib and palbociclib (FGFR and CDK4/6 inhibitor).

## Graphical abstract

**Figure:**
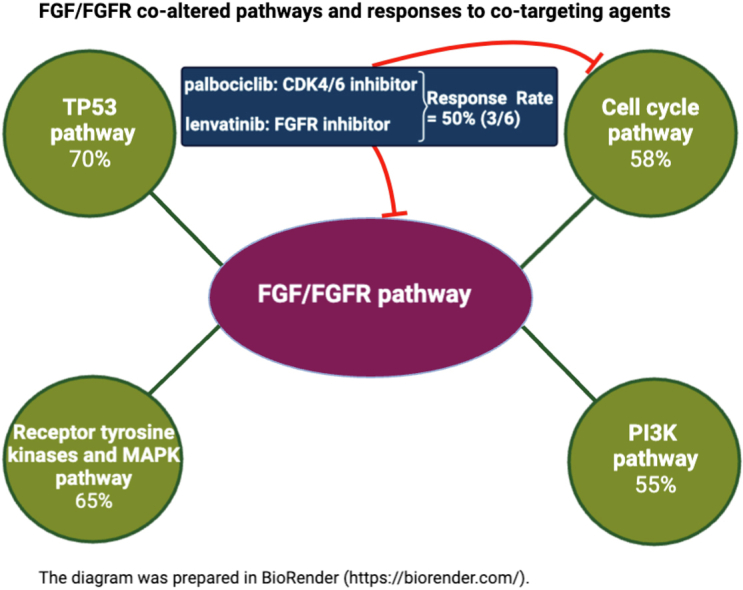

## Introduction

Fibroblast growth factor receptors (FGFRs) are highly conserved transmembrane tyrosine kinase receptors, which regulate basic biologic process such as development, differentiation, cell survival, migration, angiogenesis, and carcinogenesis.^1^ FGFR forms a family of four tyrosine kinase receptors (FGFR1-4), and one that lacks an intracellular tyrosine kinase domain (FGFR5).^2^ In humans, >20 unique ligands are identified, which are known as fibroblast growth factors (FGFs).^3^ Ligand binding to FGFR leads to intracellular phosphorylation of receptor kinase domains, a cascade of intracellular signaling, and gene transcription.^4^ Downstream signaling is triggered by intracellular receptor substrates FGFR substrate 2 (FRS2) and phospholipase Cg (PLC-g), leading to subsequent up-regulation of mitogen-activated protein kinase (MAPK), phosphoinositide 3-kinase (PI3K), and signal transducer and activator of transcription (STAT) signaling pathways.^5^

Aberrant FGFR signaling as a result of gene amplification, point mutations, and gene fusions has been observed in different tumor types; these alterations are promising targets for cancer therapeutics.^6^ An aberrantly activated FGF/FGFR signaling axis is likely to contribute to tumor growth and proliferation, promote neo-angiogenesis, and participate in acquired resistance to anticancer therapies. Using next-generation sequencing (NGS) with a review of nearly 5000 cancer patients, *FGFR* aberrations were found in 7.1% of malignancies.^7^

There are several non-selective multikinase inhibitors that target FGF/FGFR pathways. Food and Drug Administration (FDA)-approved agents include: ponatinib, regorafenib, pazopanib, and lenvatinib. *FGFR* alteration status is not required to use those medications. Recently, selective FGFR inhibitors have been investigated specifically for *FGFR*-altered solid tumors.^8^ To date, the FDA has approved erdafitinib for urothelial cancer with *FGFR2* or *FGFR3* alterations, as well as pemigatinib and infigratinib for cholangiocarcinoma with *FGFR2* fusions or other rearrangements.^9^, ^10^, ^11^

Although multiple studies have found that the aberrant FGFR signaling pathway is an attractive therapeutic target, FGFR inhibitor-based therapies do not always benefit patients, even if one selects for *FGFR*-altered cancers.^6^ The potential reasons for the variable efficacy of FGFR targeting may be related to multiple factors, but we hypothesized that, in some patients, concomitant oncogenic alterations that appear along with *FGFR* abnormalities could be associated with primary and/or secondary resistance.^7^^,^^12^^,^^13^ Targeting one specific signal in a complex network of genomic drivers may not be sufficient to control cancer progression.

We have previously shown that dual inhibition of MAPK and cell cycle pathways, when both were co-altered, could be effective, even if single-agent targeting was mostly inactive.^14^ This has been suggested to be akin to ‘whack a mole’.^15^ Furthermore, recent tumor agnostic studies demonstrate that the greater the proportion of genomic alterations targeted, the better the outcome, and studies targeting single alterations may not always show salutary effects.^16^, ^17^, ^18^, ^19^, ^20^, ^21^ Combination approaches may conceivably overcome the limitation of single-agent FGFR suppression. However, there are limited data reflecting actual clinical practice regarding matched therapy targeting both the FGFR pathway and the co-alterations.

Herein, we provide evidence that FGFR pathway alterations are associated with a poor prognosis. Even so, co-targeting FGF/FGFR axis alterations and concomitant deleterious alterations showed activity in advanced refractory malignancies.

## Materials and methods

### Analysis of 1074 patients from cBioPortal

A total of 1074 patients with *FGF*/*FGFR* alterations of *FGF3*/*4*/*19* and *FGFR1*/*2*/*3*/*4,* and 6500 patients without those *FGF*/*FGFR* alterations were analyzed from the Memorial Sloan Kettering-Integrated Mutation Profiling of Actionable Cancer Targets (MSK-IMPACT) Clinical Sequencing Cohort from cBioPortal (cBio cancer genomics portal) for cancer genomics to evaluate correlations between *FGF/FGFR* alterations and other pathway perturbations at the genomic level, as well as outcome (Supplementary Figure S1, available at https://doi.org/10.1016/j.esmoop.2022.100647).^22^

### Study population receiving FGFR-targeted treatment after MTB presentation

A total of 715 patients with malignancies and available molecular diagnostics were discussed at the (face-to-face) University of California San Diego (UCSD) Molecular Tumor Board (MTB) (Supplementary Figure S2, available at https://doi.org/10.1016/j.esmoop.2022.100647).^20^ Among these patients, 16 patients who received an FGFR inhibitor to target alterations in *FGF3*/*4*/*6/14*/*19*/*23*, *FGFR1*/*2*/3/4, and *FRS2* were identified [note: fibroblast growth factor receptor substrate 2 (FRS2) is an adaptor protein that plays a critical role in FGFR signaling].^23^

### Somatic alteration identification and annotation

Somatic alterations were identified in tumor tissues by hybrid capture-based targeted DNA sequencing using FoundationOne CDx.^24^ Somatic alterations in blood-derived cell-free DNA (cfDNA) were detected by Guardant360.^25^ Both assays apply NGS of cancer-related genes. The figure of the landscape of somatic mutations was created using the visualized data feature by the OncoPrinter tool in cBioPortal.^26^^,^^27^ Somatic mutation annotation of the biological and oncogenic effects was extracted from the OncoKB knowledge base.^28^

### Molecular matching

The MTB made recommendations to optimize molecular matching for each patient, trying to cover as many aberrant genomic alterations by cognate therapies as possible.^16^^,^^18^^,^^20^^,^^21^ Physicians, however, ultimately chose the patient’s therapy and they could decide to follow MTB recommendations or not.

### Statistical analysis

Summary statistics were used to describe patient characteristics. We evaluated progression-free survival (PFS), which was defined as the time between the start of the treatment and disease progression confirmed by imaging or clinical findings. Overall survival (OS) was measured as the time between the onset of therapy until the last follow-up. Patients without progression at the last follow-up date were censored for PFS at that date. Patients alive at last follow-up were censored for OS at that point. PFS and OS data were represented by Kaplan–Meier estimation and the survival endpoints were compared using log-rank tests. OS data from cBioPortal represents the time to metastatic/advanced disease to death or censoring as above. Hazard ratio (HR) was computed by log-rank test and Cox regression analysis. All *P* values ≤ 0.05 were considered significant. All statistical analyses were carried out using R version 4.1.1.

### Ethics statement

Our study was carried out according to the guidelines of the UCSD Institutional Review Board [Profile Related Evidence Determining Individualized Cancer Therapy (PREDICT), NCT02478931] and I-PREDICT (NCT02534675) and for any investigational interventions for which the patients consented.

Data for our study will be made available by the corresponding author upon reasonable request.

## Results

### Patients with FGF/FGFR-altered cancers have a worse prognosis than patients with wild-type FGF/FGFR

**Figure 1:**
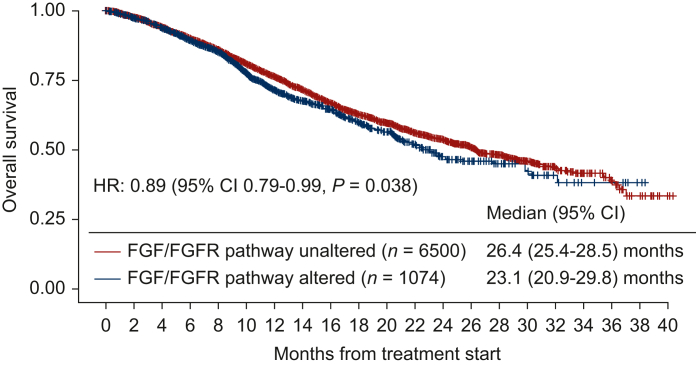
**Kaplan–Meier survival curves for overall survival stratified by *FGF/FGFR* pathway alterations (*N* = 7574, data from cBio cancer genomics portal).** Data derived from cBio cancer genomics portal, MSK-IMPACT Clinical Sequencing Cohort http://www.cbioportal.org/). Tick marks represent censored time points for patients still alive at last follow-up. Overall survival from time of metastatic/advanced disease comparing patients with *FGF/FGFR* pathway-unaltered and *FGF/FGFR* pathway-altered cancers. *FGF/FGFR* pathway alterations included *FGF3*/*4*/*19*, *FGFR1*/*2*/*3*/*4*, and *FGF6*/*23*; *FRS2* genes that were found in the UCSD cohort were not listed since they were not assessed in the MSK-IMPACT Clinical Sequencing Cohort. Overall survival analysis was based on *FGF/FGFR* alteration status. When compared to *FGF/FGFR* pathway-unaltered cases (*N* = 6500), *FGF/FGFR* pathway-altered cases (*N* = 1074) had worse overall survival with a HR of 0.89 (95% CI 0.79-0.99, *P* = 0.038). CI, confidence interval; HR, hazard ratio; MSK-IMPACT, Memorial Sloan Kettering-Integrated Mutation Profiling of Actionable Cancer Targets; UCSD, University of California San Diego.

When compared to patients with *FGF/FGFR*-unaltered cancers (*N* = 6500), patients who harbored cancers with *FGF/FGFR* gene alterations (*N* = 1074) had significantly shorter OS [median OS: 26.4 months versus 23.1 months, HR: 0.89, 95% confidence interval (CI): 0.79-0.99, *P* = 0.038; Figure 1].

### FGF/FGFR pathway alterations were frequently accompanied by alterations in other signaling pathways

**Figure 2:**
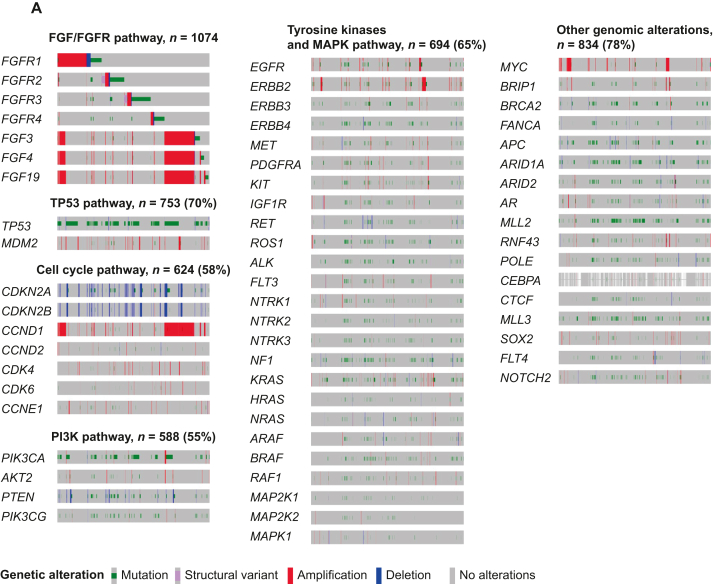

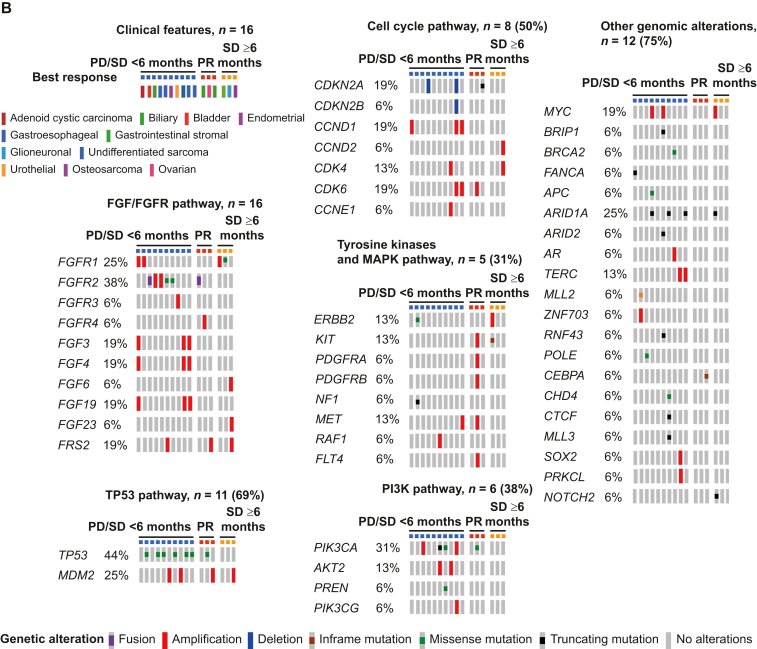
**Summary of selected co-altered oncogenic genes along with*****FGF/FGFR*****pathway alterations.** (A) Integrated view of oncogenic genes co-occurring with *FGF/FGFR* alterations from cBio cancer genomics portal (*N* = 1074, MSK-IMPACT Clinical Sequencing Cohort, data from cBio cancer genomics portal, http://www.cbioportal.org/). Each column denotes an individual patient, and each row displays a gene. Genetic alterations are color coded by the type of alterations. Co-alterations in oncogenic pathways were observed in *TP53* pathway genes (70%), cell cycle–associated genes (58%), receptor tyrosine kinases and *MAPK* pathway-associated genes (65%), PI3K signaling-associated genes (55%), and other genomic alteration genes (78%). *FGF6*, *FGF23*, *FRS2*, *TERC*, *ZNF703*, *CHD4*, and *PRKCL* genes that were found in the UCSD cohort were not listed since they were not assessed in the MSK-IMPACT Clinical Sequencing Cohort. Selected genes of relevant pathways were listed. The diagram was prepared in OncoPrinter tool using cBio cancer genomics portal (http://www.cbioportal.org/). (B) Integrated view of co-occurring somatic oncogenic alterations in 16 cases with *FGF/FGFR* pathway alterations who received FGFR inhibitors (*N* = 16, UCSD patients). Each column denotes an individual patient, and each row displays a gene. Genetic alterations are color coded by the type of alterations. Co-alterations in oncogenic pathways were observed in *TP53* pathway genes (71%), cell cycle–associated genes (53%), receptor tyrosine kinases and *MAPK* pathway-associated genes (35%), PI3K signaling-associated genes (41%), and other genomic alteration genes (76%). The diagram was prepared in OncoPrinter tool using cBio cancer genomics portal (http://www.cbioportal.org/). MSK-IMPACT, Memorial Sloan Kettering-Integrated Mutation Profiling of Actionable Cancer Targets; UCSD, University of California San Diego.

When interrogating the cBioPortal cohort (*N* = 1074), the most frequently co-altered pathways/genes were: *TP53* [70% (753/1074)]; cell cycle [58% (624/1074)]; PI3K pathway [55% (588/1074)]; receptor tyrosine kinases and MAPK pathway [65% (694/1074)]; and other genomic alterations [78% (834/1074) patients] (Figure 2A). Only 65 patients of 1074 (6.1%) had no co-alterations accompanying their *FGF/FGFR* axis genomic abnormalities.

The landscape of FGF/FGFR pathway and co-occurring alternations in the UCSD cohort of FGFR inhibitor-treated patients (*N* = 16) is shown in Figure 2B and in Tables 1 and 2 and is similar to those derived from the larger cBioPortal cohort with regard to the commonly co-altered genes/pathways accompanying *FGF/FGFR* axis aberrations. The median number of alterations in the *FGF/FGFR* genes was 1 (range, 1-4). Alterations in *FGF3*, *FGF4*, *FGF19*, and *CCND1* often co-occurred, probably because they reside on the same amplicon on 11q13. Alterations in *MDM2* and *FRS2* were mostly concomitant, probably because they reside on the same amplicon on 12q15. Among UCSD patients with *FGF/FGFR* pathway alterations, a wide variety of gene co-alterations were seen (Figure 2B). The most frequently co-altered pathways/genes were: *TP53* [69% (11/16)]; cell cycle [50% (8/16)]; PI3K pathway [38% (6/16)]; receptor tyrosine kinases and MAPK pathway [31% (5/16)]; and other genomic alterations [75% (12/16)] patients.

**Table 1:** Characteristics of patients with *FGF/FGFR* pathway gene alterations who received FGFR inhibitor-based therapies (*N* = 16; UCSD cohort)

Patient characteristics	*N* = 16
Median age^a^ (range), year	61 (21-82)
Sex, *n* (%)	
Men	6 (37.5)
Women	10 (62.5)
Type of cancer, *n* (%)	
Gastroesophageal	4 (25.0)
Biliary	3 (18.8)
Bladder	1 (6.3)
Urothelial	1 (6.3)
Ovarian	1 (6.3)
Endometrial	1 (6.3)
Glioneuronal	1 (6.3)
Osteosarcoma	1 (6.3)
Gastrointestinal stromal	1 (6.3)
Adenoid cystic carcinoma	1 (6.3)
Undifferentiated sarcoma	1 (6.3)
*FGF/FGFR* alterations,^b^*n* (%)	
Amplification	12 (75.0)
Single-nucleotide variant	3 (18.9)
Fusion	2 (12.5)
Type of FGFR inhibitor, *n* (%)^c^	
Lenvatinib	12 (75.0)
Ponatinib	2 (12.5)
Pazopanib	1 (6.3)
Infigratinib	1 (6.3)
Number of molecularly matched drugs, *n* (%)	
Monotherapy	5 (31.3)
≥Two matched therapies	11 (68.8)
Best response, *n* (%)	
Progressive disease or stable disease <6 months	10 (62.5)
Stable disease ≥6 months	3 (18.8)
Partial response	3 (18.8)
Median number of lines of prior therapies (range)	2 (1-12)
Median number of deleterious co-alterations (range)^d^	5 (0-10)
Median progression-free survival, month (range)	4.6 (2.8-51.7)
Median overall survival, month (range)	13.5 (1.2-51.7)

FGFR, fibroblast growth factor receptor; UCSD, University of California San Diego.

a Age at the time of metastatic/locally advanced disease.

b One patient had both amplification and a single-nucleotide variant.

c See Supplementary Table S1, available at https://doi.org/10.1016/j.esmoop.2022.100647 for 50% inhibitory concentrations (IC_50_) of these drugs.

d Variants of unknown significance excluded.

**Table 2:** Clinical and molecular characteristics as well as clinical outcomes of patients treated with FGFR inhibitor-based therapies (*N* = 16)

ID	Age, years	Sex	Diagnosis	Molecular characteristics (laboratory vendor, source)	PD-L1 CPS^a^	TMB (muts/Mb)	Treatment regimen	Number of prior line of therapies	PFS (month)	OS (month)	Best response
1	37	F	Gastroesophageal cancer	(FM, tissue) *FGFR2* amplification *CDKN2A* loss *MYC* amplification *APC* I1307K *ARID1A* P2139fs∗62 *TP53* F113C	Unknown	Unknown	Ponatinib	6	2.3	2.3	PD
2	78	M	Bladder cancer	(FM, tissue) *FGFR1* amplification *NF1* Q1218∗ *TP53* R267G *ERBB2* I767M *MLL2* P3668fs∗5, splice site 177-1G>T *ZNF703* amplification	Unknown	Unknown	Pazopanib	3	1.2	1.2	PD
3	72	M	Urothelial cancer	(FM, tissue) *FRS2* amplification *AKT2* amplification *BRIP1* truncation *PIK3CA* H450_V461>GS *RAF1* amplification *MDM*2 amplification *MYC* amplification *RNF43* S262∗ *ARID2* S889∗ (GH, blood) *FGFR2* V516L, G131R	Unknown	Unknown	Lenvatinib, olaparib	2	5.8	10.2	SD <6 months
**4**	**33**	**F**	**Biliary cancer**	**(FM, tissue)** ***FGFR2-BICC1* fusion**	**Unknown**	**Unknown**	**Infigratinib**	**2**	**21.6+**	**21.6+**	**PR**
5	47	F	Biliary cancer	(FM, tissue) *FGFR2-BICC2* fusion *POLE* R446Q (GH, blood) *PIK3CA* amplification	Unknown	2.4	Lenvatinib, everolimus	2	3.7	3.7	SD <6 months
**6**	**81**	**M**	**Biliary cancer**	**(FM, tissue)** ***FRS2* amplification** ***MDM2* amplification** ***CDKN2A* p16INK4a R80∗,** **p14ARF P94L** ***CEBPA* G103_G104del**	**Unknown**	**Unknown**	**Lenvatinib, palbociclib**	**1**	**11.8**	**12.0**	**PR**
**7**	**30**	**F**	**Osteosarcoma**	**(FM, tissue)** ***FGF23* amplification** ***FGF6* amplification** ***FRS2* amplification** ***CDK4* amplification** ***CCND2* amplification** ***MDM2* amplification**	**Unknown**	**3**	**Lenvatinib, palbociclib**	**4**	**51.7+**	**51.7+**	**PR (per PERCIST)** **Note: At 32 months, the patient had a new lesion that was resected; she has continued on therapy, doing well at 51.7+ months**
8	60	M	Gastroesophageal cancer	(FM, tissue) *FGF19* amplification *FGF3* amplification *FGF4* amplification *CCND1* amplification *CDK6* amplification *MET* amplification *ARID1A* R1276∗ *TERC* amplification *TP53* P278L	10	8	Lenvatinib, palbociclib, nivolumab	2	0.9	2.5+	PD
**9**	**21**	**F**	**Glioneuronal tumor**	**(FM, tissue)** ***FGFR1* K656E**	**Unknown**	**2**	**Lenvatinib**	**2**	**15.1**	**30.6+**	**SD ≥6 months**
10	73	F	Endometrial cancer	(FM, tissue) *FGFR2* N549K *PIK3CA* G1049R *PTEN* K125N *ARID1A* Q2115fs∗33 *CHD4* R975H *CTCF* S282fs∗21 *MLL3* S2123∗ *TP53* Y163C	2	1	Lenvatinib, everolimus	1	3.2	22.1	PD
11	54	M	Gastroesophageal cancer	(FM, tissue) *FGF19* amplification *FGF3* amplification *FGF4* amplification *CCND1* amplification *CDK6* amplification *CDKN2A/B* loss *PIK3CA* amplification *SOX2* amplification *PIK3CG* amplification *PRKCI* amplification *TERC* amplification *TP53* G245D	1	7	Lenvatinib, palbociclib, nivolumab	1	2.7	2.7	PD
12	82	M	Gastroesophageal cancer	(FM, tissue) *FGFR2* amplification *TP53* A159V	Unknown	4	Lenvatinib	1	4.7	6.5	SD <6 months
**13**	**63**	**F**	**Ovarian cancer**	**(FM, tissue)** ***FGFR4* amplification** ***FLT4* amplification** ***PDGFRB* amplification** ***CDK6* amplification** ***TP53* K132R** **(GH, blood)** ***TP53* K132, K120M** ***PIK3CA* E545K** ***MET* amplification** ***PDGFRA* amplification** ***KIT* amplification**	**Unknown**	**6**	**Lenvatinib, palbociclib**	**12**	**8.9**	**17.6+**	**PR**
**14**	**72**	**F**	**Gastrointestinal stromal tumor**	**(FM, tissue)** ***KIT* K558_E562del, N822K, V654A** ***ARID1A* truncation** ***NOTCH2* P6fs∗27** **(GH, blood)** ***FGFR1* amplification** ***MYC* amplification** ***ERBB2* amplification**	**Unknown**	**7**	**Lenvatinib, pembrolizumab**	**4**	**13.1+**	**13.1+**	**SD ≥6 months**
15	61	F	Adenoid cystic carcinoma	(FM, tissue) *FGF19* amplification *FGF3* amplification *FGF4* amplification *CCND1* amplification *FANCA* F1263del (GH, blood) *FGFR1* amplification	Unknown	Unknown	Lenvatinib, palbocicib	4	2.8	2.8+	PD
16	47	F	Undifferentiated sarcoma	(FM, tissue) *FGFR3* amplification *AKT2* amplification *BRCA2* R1190W *CCNE1* amplification *CDK4* amplification *MDM2* amplification *AR* amplification	Unknown	Unknown	Pazopanib, everolimus	1	4.4	15.2	SD <6 months

CPS, combined positive score; FM, Foundation Medicine; GH, Guardant Health; muts/Mb, mutations per megabase; PD progressive disease; PD-L1, programmed death ligand-1; PR, partial response; SD, stable disease; TMB, tumor mutational burden.

Cases with SD >6 months/PR were highlighted in bold.

a PD-L1 was assessed with the 22C3 antibody.

### Patients harboring cancers with FGF/FGFR pathway gene alterations accompanied by TP53 pathway or cell cycle pathway alterations had worse overall survival compared to patients with FGF/FGFR-altered cancers without those co-alterations

**Figure 3:**
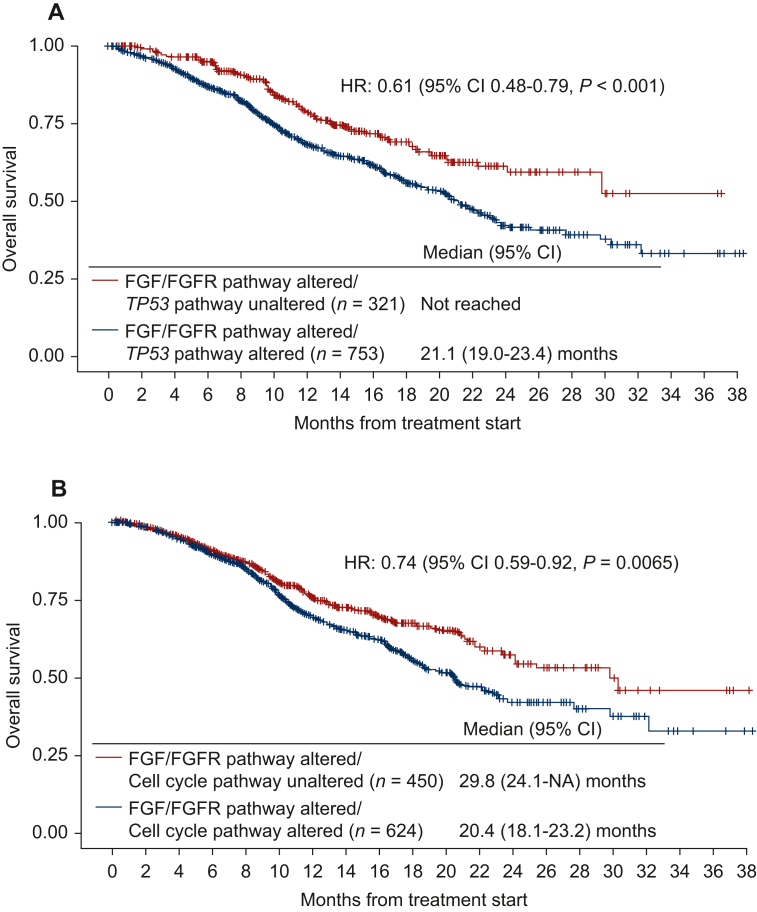

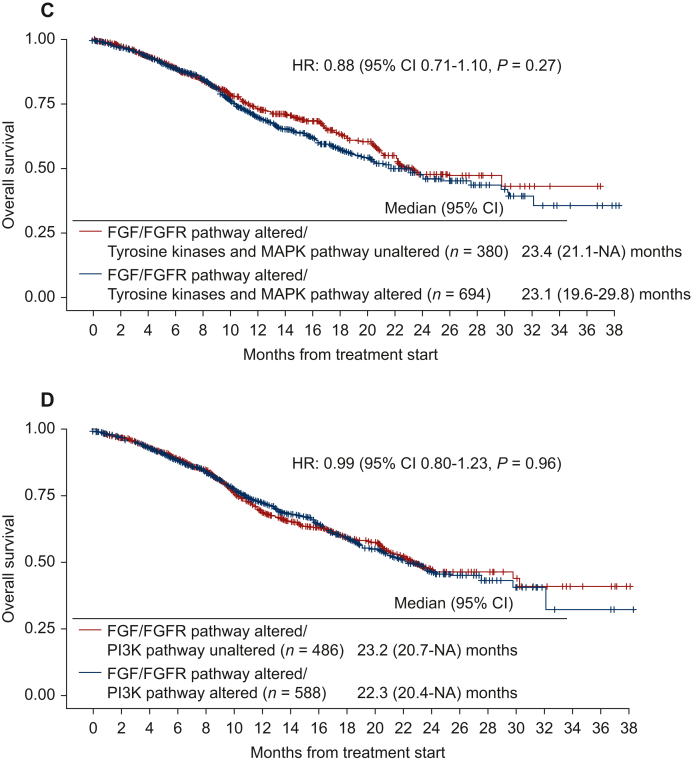
**Overallsurvival among patients with *FGF/FGFR* pathway alterations stratified by co-occurring alterations in *TP53* pathway (*TP53*, *MDM2*), cell cycle pathway (*CDKN2A*, *CDKN2B*, *CCND1*, *CCND2*, *CDK4*, *CDK6*), receptor tyrosine kinases and MAPK pathway (*EGFR*, *ERBB2*, *ERBB3*, *ERBB4*, *MET*, *PDGFRA*, *KIT*, *IGF1R*, *RET*, *ROS1*, *ALK*, *FLT3*, *NTRK1*, *NTRK2*, *NTRK3*, *NF1*, *KRAS*, *HRAS*, *NRAS*, *ARAF*, *BRAF*, *RAF1*, *MAP2K1*, *MAP2K2*, *MAPK1*), and PI3K pathway (*PIK3CA*, *AKT2*, *PTEN*, *PIK3CG* (*N* = 1074, data from cBio cancer genomics portal).** Data derived from cBioPortal cancer genomics, MSK-IMPACT Clinical Sequencing Cohort. (A) Overall survival comparing the impact of *FGF/FGFR* and *TP53* pathway alterations. When compared to *FGF/FGFR* pathway-altered/*TP53* pathway-unaltered cases (*N* = 321), *FGF/FGFR* pathway-altered/*TP53* pathway-altered cases (*N* = 753) had significantly worse overall survival (HR of 0.61, 95% CI 0.48-0.79, *P* < 0.001). (B) Overall survival comparing the impact of *FGF/FGFR* and cell cycle pathway alterations. When compared to *FGF/FGFR* pathway-altered/cell cycle pathway-unaltered cases (*N* = 450), *FGF/FGFR* pathway-altered/cell cycle pathway-altered cases (*N* = 624) had significantly worse overall survival (HR of 0.74, 95% CI 0.59-0.92, *P* = 0.007). (C) Overall survival comparing the impact of *FGF/FGFR* and receptor tyrosine kinases and MAPK pathway alterations. When compared to *FGF/FGFR* pathway-altered/receptor tyrosine kinases and MAPK pathway-unaltered cases (*N* = 380*), FGF/FGFR* pathway-altered/receptor tyrosine kinases and MAPK pathway-altered cases (*N* = 694) did not demonstrate a significant difference in overall survival (HR of 0.88, 95% CI 0.71-1.10, *P* = 0.27). (D) Overall survival comparing the impact of *FGF/FGFR* and PI3K pathway alterations. When compared to *FGF/FGFR* pathway-altered/PI3K pathway-unaltered cases (*N* = 486), *FGF/FGFR* pathway-altered/PI3K pathway-altered cases (*N* = 588) did not demonstrate a significant difference in overall survival (HR of 0.99, 95% CI 0.80-1.23, *P* = 0.96). See Figure 2A and B for the list of selected genes associated with the pathway alterations. CI, confidence interval; HR, hazard ratio; NA, not available.

Interrogating all 1074 individuals with *FGF/FGFR*-altered tumors, those harboring alterations in both *FGF/FGFR* and in the *TP53* pathway (HR: 0.61, 95% CI 0.48-0.79, *P* = 0.0001; Figure 3A) or in the cell cycle pathway (along with the *FGF/FGFR* genes) (HR: 0.74, 95% CI 0.59-0.92, *P* = 0.0065; Figure 3B) had significantly shorter OS when compared to patients with *FGF/FGFR*-altered cancers without those co-altered anomalies. However, patients who had both *FGF/FGFR* abnormalities and co-alterations in receptor tyrosine kinases or MAPK or PI3K pathway showed no significant difference in OS when compared to patients with cancers harboring *FGF/FGFR* pathway alterations without those co-altered pathways (Figure 3C and D).

### Characteristics of patients with FGF/FGFR-altered cancers who received FGFR inhibitor therapy

Sixteen patients with *FGF/FGFR* pathway alterations received FGFR inhibitors (Supplementary Figure S2, available at https://doi.org/10.1016/j.esmoop.2022.100647) at UCSD. Demographics and patient characteristics are shown in Table 1. The median age was 61 years. Six patients (37.5%) were men. The most common diagnosis was gastroesophageal cancer [25.0% (4/16)], followed by biliary cancer [18.8% (3/16)]. *FGF/FGFR* amplification occurred in 12 patients, single-nucleotide variant (SNV) in three patients, and fusions in two patients (Table 1). One patient had both amplification and SNV. Five patients (31%) received FGFR inhibitors as monotherapy; 11 patients (69%) received a customized combination. The median number of prior therapies was 2 (range, 1-12). The median number of co-alterations, excluding *FGF/FGFR* alterations, was 5 (range, 0-10). The median PFS and OS were 4.6 months and 13.5 months, respectively.

### FGFR inhibitors have activity in the UCSD cohort of patients with FGF/FGFR axis alterations, especially in combination with cyclin inhibitors when cyclin-activating genes are co-altered

Overall, 16 patients with *FGF/FGFR* alterations were treated with drugs with potent FGFR inhibitory activity (5 with monotherapy and 11 with an FGFR inhibitor combined with one or more other drugs that matched co-activated signals) (Supplementary Tables S1 and S2, available at https://doi.org/10.1016/j.esmoop.2022.100647). Partial responses (PRs) were seen in four patients (9, 12, and 22+, and 52+ months) and an additional two patients achieved stable disease (SD) ≥6 months (13+ and 15 months). Therefore, 6 of 16 patients attained clinical benefit (SD ≥ 6 months/PR) [clinical benefit rate (CBR): 38%] (Table 2).

Five patients received monotherapy with an FGFR inhibitor; two achieved SD ≥ 6 months/PR—both with single alterations on NGS [*FGFR2* fusion (biliary tract cancer treated with infigratinib) and *FGFR* K656E mutation (glioneuronal tumor treated with lenvatinib)]; the other patients, who did not respond, had pathogenic genomic co-alterations in their cancers.

Four of 11 patients (36%) who received customized combination therapy achieved SD ≥ 6 months/PR; all of these patients had tumors harboring pathogenic co-alterations in addition to their FGF/FGFR genomic anomalies*.* These four patients had malignancies as follows (Table 2 and Supplementary Table S2, available at https://doi.org/10.1016/j.esmoop.2022.100647): (i) a biliary cancer with an *FRS2* amplification and *CDKN2A* alteration treated with a combination of the FGFR inhibitor lenvatinib and the CDK4/6 inhibitor palbociclib (achieving a PR for ∼12 months); (ii) a patient with osteosarcoma whose tumor harbored amplifications in *FGF23*, *FGF6*, *FRS2* as well as *CDK4* and *CCND2* amplifications [treated with lenvatinib and palbociclib with an ongoing PERCIST 1.0 (PET) response at 52+ months^29^]; (iii) an ovarian cancer harboring *FGFR4* and *CDK6* amplification (also treated with lenvatinib and palbociclib with a PR that lasted ∼9 months); and (iv) a gastrointestinal stromal tumor (status after four prior systemic therapies) with *FGFR1* amplification as well as *MYC* and *ERBB2* amplification, and *KIT, ARID1A*, and *NOTCH2* mutations treated with lenvatinib and pembrolizumab (*ARID1A* alterations may induce sensitivity to immune checkpoint blockade^30^) (achieving SD ongoing at 13.1+ months).

Importantly, six patients received a combination of drugs that included the CDK4/6 inhibitor palbociclib (administered because of the presence of alterations in the cyclin pathway) and the multi-tyrosine kinase inhibitor (including FGFR inhibitor) lenvatinib. Three of the six patients (50%) achieved a PR (lasting 9, 12, and 52+ months) (Table 2). The doses used were palbociclib 75 mg p.o. 3 weeks on/1 week off (approved dose = 125 mg p.o. 3 weeks on and 1 week off) and lenvatinib 10 mg p.o. daily (approved dose = 24 mg p.o. daily). In each case, the lower modified dose was the starting dose, and because tolerability was good and efficacy was seen, the doses were not further titrated upwards. The most common side effects in these patients were grade 1-2 rash and fatigue, which were manageable without dose adjustments. There was one patient who experienced grade 3 neutropenia and required dose modification of palbociclib (dose decreased to 75 mg p.o. 1 week on and 1 week off).

## Discussion

Herein we report prognostic and predictive observations related to the presence of *FGF/FGF*R genomic alterations in a group of pan-cancer patients. Our results indicate that *FGF/FGFR* axis alterations portend a poor prognosis, as reflected by the observation that patients whose malignancies harbor *FGF/FGFR* genomic alterations have significantly shorter survival than those with malignancies that are wild type for *FGF/FGFR*. These results are consistent with prior observations of worse prognosis in patients whose tumors harbor *FGFR* amplification.^31^^,^^32^

Importantly, 94% of 1074 cancers harbored genomic co-alterations in addition to the *FGF/FGFR* genomic abnormalities. The most common co-altered pathways/genes were in the *TP53* axis (*TP53* or *MDM2* genes) (70% of cancers); cell cycle (58%); PI3K pathway (55%); and receptor tyrosine kinases and MAPK pathway (65%). Additional genomic alterations were observed in 78% of patients (Figure 2A). The presence of *FGF/FGFR* axis genomic alterations along with *TP53/MDM2* or cell cycle pathway alterations correlated with worse OS compared to individuals with *FGF/FGFR-*altered cancers without those co-alterations. Co-alterations in receptor tyrosine kinases or MAPK or PI3K pathway did not show prognostic significance.

Sixteen patients with diverse malignancies presented at the UCSD face-to-face MTB^20^ had *FGF/FGFR* pathway genomic alterations and were treated with cognate FGFR inhibitors, either alone or together with agents that targeted pathogenic co-alterations. Targeting FGFR signaling alone or together with co-alterations achieved a clinical benefit rate (SD ≥ 6 months/PR/CR) of 38% (6/16) and a median PFS of 14 months among those six patients. Most of the tumors [88% (14/16)] had genomic co-alterations along with the *FGF/FGFR* pathway abnormalities. These co-alterations could conceivably be associated with primary resistance to FGFR inhibition. Indeed, it has been recognized that targeting *FGF/FGFR* pathway alterations is complicated.^13^ Some challenges include differences in antitumor activity among different cancer diagnoses, and variable responses depending on the type of *FGFR* alterations [fusions and mutations have been reported to achieve higher clinical benefit from FGFR-tyrosine kinase inhibitors (TKIs) when compared to amplification].^8^^,^^33^ Moreover, multiple different mechanisms for primary and acquired resistance to FGFR-targeted therapies have been revealed.^34^, ^35^, ^36^

We hypothesized that resistance to FGFR inhibitors in *FGF/FGFR*-altered cancers could be due, in some cases, to genomic complexity with co-alterations that differ from patient to patient. In our cohort, in line with our hypothesis, 14 of our 16 patients had ≥1 potentially important molecular co-alterations in their cancer. A similar phenomenon was observed in the MSK-IMPACT Clinical Sequencing Cohort. The median number of deleterious co-alterations, excluding *FGF/FGFR* alterations, in the UCSD cohort was 5 (range, 0-10).

With regard to FGFR inhibitor therapy, altogether 6 out of 16 of our patients derived clinical benefit [SD ≥ 6 months (*N* = 2)/PR (*N* = 4)]. The two patients with *FGFR* alterations (a fusion and a mutation) and no co-alterations responded to single-agent *FGFR* inhibitors. The other four responders received a combination regimen that targeted FGFR and one or more of the co-alterations.

The most common combination utilized included palbociclib (CDK4/6 inhibitor) (given due to cyclin pathway genomic abnormalities) and the potent multi-tyrosine kinase FGFR inhibitor lenvatinib (given to six patients). Three of the six patients (50%) attained a PR (duration 9, 12, and 52+ months). The doses used were palbociclib 75 mg p.o. for 3 weeks on and 1 week off (approved dose = 125 mg p.o. 3 weeks on and 1 week off) and lenvatinib 10 mg p.o. daily (approved dose = 24 mg p.o. daily). In each case, there were no serious adverse events and efficacy was observed in half of the patients, despite the fact that doses were substantially lower than those approved by the FDA for the individual drugs.

It has been reported that tumors with *FGFR* fusions appear to respond to FGFR inhibition.^13^^,^^37^^,^^38^ In our cohort, there were two patients with an *FGFR* fusion. One patient attained a durable PR, and the other patient did not respond; both patients had biliary cancers. There were several potential reasons for different clinical outcomes. First, the patient who achieved a PR had no co-alterations, while the other patient had concomitant molecular alterations, including in the PI3K pathway, a signal transduction pathway downstream of FGFR pathway.^39^ Co-occurrence of FGF/FGFR and PI3K pathway alterations could imply cooperation as potential dual oncogenic drivers.^40^, ^41^, ^42^ Therefore, an FGFR inhibitor as a monotherapy would not disrupt oncogenic signaling driven by PI3K pathway alterations. Preclinical studies have reported a high synergistic activity between PI3K and FGFR inhibitors.^43^, ^44^, ^45^ However, the early phase combination trial was challenging because of difficulty with tolerability with the high rates of treatment interruptions and dose modification.^46^ Another factor may be that, while lenvatinib is a potent FGFR inhibitor, infigratinib (which was the drug administered to the patient who responded) has an even lower IC50 for FGFR (Supplementary Table S1, available at https://doi.org/10.1016/j.esmoop.2022.100647). Finally, the fusion *FGFR2-BICC1* in the case of the patient who attained a PR is known to be a typical oncogenic driver, while FGFR2-BICC2 in the other patient may not be as clear-cut.^8^

There are several limitations to the current report. Firstly, the therapeutic arm of the study has a small sample size and a variety of histologies (though the latter may also suggest generalizability of results). Secondly, molecular characteristics of tumors could evolve, particularly with therapeutic pressure, and serial molecular follow-up was not available. Future investigations may require molecular sequential analysis, such as monitoring by circulating tumor cell-free DNA analysis. Thirdly, some *FGF/FGFR* pathway alterations might not be pivotal for cancer cells, and targeting other accompanied signaling pathways might have a greater effect. Finally, most of the FGFR inhibitors used in this study were not selective to the *FGF/FGFR* pathway. However, despite these limitations, the current research targeting *FGF/FGFR* signaling in real-world practice provides evidence for the importance of impacting the products of co-occurring alterations, especially in the cyclin pathway.

In conclusion, we demonstrate that *FGF/FGFR-*altered tumors have a poor prognosis and frequent co-alterations in several important pathways. Co-targeting of cyclin and *FGF/FGFR* alterations with the CDK4/6 inhibitor palbociclib and the FGFR inhibitor lenvatinib can be carried out safely, with responses seen in three of six patients, including ongoing benefit in a patient with refractory osteosarcoma who continues to do well at 52+ months. Further prospective evaluation of this strategy is warranted in patients whose tumors harbor *FGF/FGFR* pathway alterations.

## Funding

None declared.

## Disclosure

**SI** received speaker’s fee from Roche, Chugai pharmaceutical Co., Merck, AstraZeneca, MSD, Taho pharmaceutical Co., Novartis, Boehringer Ingelheim, Act Med, Bayer, Takeda, Guardant Health, IQVIA, and Cannon Medical. He received research funding from ACT Genomics, Cannon Medical, and Hitachi Systems. **SK** serves as a consultant for Medpace, Foundation Medicine, NeoGenomics, and CureMatch. He receives speaker’s fees from Roche and Bayer, and advisory board for Pfizer. He has research funding from ACT Genomics, Sysmex, Konica Minolta, OmniSeq, and Personalis. **JKS** receives research funding from Novartis Pharmaceuticals, Amgen Pharmaceuticals, and Foundation Medicine; consultant fees from Grand Rounds, Loxo, and Deciphera; and speaker’s fees from Roche and Deciphera. He also owns stocks in Personalis. **RK** has received research funding from Biological Dynamics, Boehringer Ingelheim, Debiopharm, Foundation Medicine, Genentech, Grifols, Guardant, Incyte, Konica Minolta, Medimmune, Merck Serono, Omniseq, Pfizer, Sequenom, Takeda, and TopAlliance; as well as consultant and/or speaker fees and/or advisory board for Actuate Therapeutics, AstraZeneca, Bicara Therapeutics, Biological Dynamics, Daiichi, EISAI, EOM Pharmaceuticals, Iylon, Merck, NeoGenomics, Neomed, Pfizer, Prosperdtx, Roche, TD2/Volastra, Turning Point Therapeutics, X-Biotech; has an equity interest in CureMatch Inc., CureMetrix, and IDbyDNA; serves on the Board of CureMatch and CureMetrix, and is a co-founder of CureMatch. All other authors have declared no conflicts of interest.
