# Supplementary material for: Targeting the FGF/FGFR axis and its co-alteration allies
Source: ESMO Open. 2022 Nov 29;7(6):100647. doi: 10.1016/j.esmoop.2022.100647 (PMC9808461; doi:10.1016/j.esmoop.2022.100647)

Supplementary Table S1. Summary of FGFR inhibitors used in the UCSD cohort (N = 16): IC_{50}

	Target IC_{50}*
Lenvatinib	VEGFR1 (4.7 nM), VEGFR2 (3 nM), KIT (85 nM), PDGFRα (29 nM), FGFR1-4 (27–61 nM)[1]
Ponatinib	VEGFR2 (1.5 nM), KIT (12.5 nM), PDGFRα (1.1 nM), FGFR1-4 (2.2–18.2 nM)[2]
Pazopanib	VEGFR2 (8 nM), KIT (2.6 nM), PDGFR β (3 nM), FGFR1-3 (84 nM)[3,4]
Infigratinib	FGFR1 (1.1 nM), FGFR2 (1.0 nM), FGFR3 (2.0 nM), FGFR4 (61 nM)[5]

*Values listed were obtained in cell-free assays.
Abbreviations: IC_{50} = 50% inhibitory concentration

References for Supplementary Table S1

1. Hussein Z, Mizuo H, Hayato S, Namiki M, Shumaker R. Clinical Pharmacokinetic and Pharmacodynamic Profile of Lenvatinib, an Orally Active, Small-Molecule, Multitargeted Tyrosine Kinase Inhibitor. *Eur J Drug Metab Pharmacokinet*. 2017;42(6):903-914.

2. O'Hare T, Shakespeare WC, Zhu X, et al. AP24534, a Pan-BCR-ABL Inhibitor for Chronic Myeloid Leukemia, Potently Inhibits the T315I Mutant and Overcomes Mutation-Based Resistance. *Cancer Cell*. 2009;16(5):401-412.

3. FDA. PHARMACOLOGY/TOXICOLOGY REVIEW AND EVALUATION (Pazopanib). Accessed September 23, 2021. https://www.accessdata.fda.gov/drugsatfda_docs/nda/2009/022465s000_PharmR.pdf

4. Roskoski R. The role of fibroblast growth factor receptor (FGFR) protein-tyrosine kinase inhibitors in the treatment of cancers including those of the urinary bladder. *Pharmacol Res*. 2020;151:104567.

5. FDA. Highlights of prescribing information (TRUSELTIQ). Accessed September 22, 2021. https://www.accessdata.fda.gov/drugsatfda_docs/label/2021/214622s000lbl.pdf

Supplementary Table S2. Rationale for suggested therapies with FGFR inhibitor-based therapies (N = 16)

ID	Diagnosis	Molecular Characteristics (Laboratory vendor, source)	Treatment Regimen	Stable disease ≥6 months or PR/CR	Rationale for therapy	References
1	Gastroesophageal cancer	(FM, tissue)<br>*FGFR2* amplification<br>*CDKN2A* loss<br>*MYC* amplification<br>*APC* I1307K<br>*ARID1A* P2139fs*62<br>*TP53* F113C	Ponatinib	No	Ponatinib is a potent FGFR inhibitor (**Supplemental Table S1**).	
2	Bladder cancer	(FM, tissue)<br>*FGFR1* amplification<br>*NF1* Q1218*<br>*TP53* R267G<br>*ERBB2* I767M<br>*MLL2* P3668fs*5, splice site 177-1G>T<br>*ZNF703* amplification	Pazopanib	No	Pazopanib is a potent FGFR inhibitor (**Supplemental Table S1**).	
3	Urothelial cancer	(FM, tissue)<br>*FRS2* amplification<br>*AKT2* amplification<br>*BRIP1* truncation<br>*PIK3CA* H450_V461>GS<br>*RAF1* amplification<br>*MDM*2 amplification<br>*MYC* amplification<br>*RNF43* S262*<br>*ARID2* S889*<br><br>(GH, blood)<br>*FGFR2* V516L, G131R	Lenvatinib, Olaparib	No	・Lenvatinib is a potent FGFR inhibitor (**Supplemental Table S1**).<br>・BRIP1 inactivation can lead to homologous recombination repair deficiency, which may be targetable with PARP inhibitors such as olaparib.	[1,2]
4	Biliary cancer	(FM, tissue)<br>*FGFR2-BICC1* fusion	Infigratinib	Yes	Infigratinib is a potent selective FGFR inhibitor (**Supplemental Table S1**).	
5	Biliary cancer	(FM, tissue)<br>*FGFR2-BICC2* fusion<br>*POLE* R446Q<br><br>(GH, blood)<br>*PIK3CA* amplification	Lenvatinib, Everolimus	No	・Lenvatinib is a potent FGFR inhibitor (**Supplemental Table S1**).<br>・*PIK3CA* amplification can activate PI3K pathway, which can be targeted with mTOR inhibitor (everolimus).	[3,4]
6	Biliary cancer	(FM, tissue)<br>*FRS2* amplification<br>*MDM2* amplification<br>*CDKN2A* p16INK4a R80*, p14ARF P94L<br>*CEBPA* G103_G104del	Lenvatinib, Palbociclib	Yes	・Lenvatinib is a potent FGFR inhibitor (**Supplemental Table S1**).<br>・*CDKN2A* alteration can activate CDK4/6 which can be targeted with CDK4/6 inhibitor (palbociclib).	[5,6]
7	Osteosarcoma	(FM, tissue)<br>*FGF23* amplification<br>*FGF6* amplification<br>*FRS2* amplification<br>*CDK4* amplification<br>*CCND2* amplification<br>*MDM2* amplification	Lenvatinib, Palbociclib	Yes	・Lenvatinib is a potent FGFR inhibitor (**Supplemental Table S1**).<br>・*CDK4* and *CCND2* amplification may be targetable with CDK4/6 inhibitor (palbociclib).	[7]
8	Gastroesophageal cancer	(FM, tissue)<br>*FGF19* amplification<br>*FGF3* amplification<br>*FGF4* amplification<br>*CCND1* amplification<br>*CDK6* amplification<br>*MET* amplification<br>*ARID1A* R1276*<br>*TERC* amplification<br>*TP53* P278L<br>TMB: intermediate (8Muts/Mb)	Lenvatinib, Palbociclib, Nivolumab	No	・Lenvatinib is a potent FGFR inhibitor (**Supplemental Table S1**).<br>・*CDK6* and *CCND1* amplification can be targeted with CDK4/6 inhibitor (palbociclib).<br>・Patients with intermediate TMB could benefit from anti-PD-L1 inhibitor (nivolumab).	[7,8]
9	Glioneuronal tumor	(FM, tissue)<br>*FGFR1* K656E	Lenvatinib	Yes	・Lenvatinib is a potent FGFR inhibitor (**Supplemental Table S1**).	

10	Endometrial cancer	(FM, tissue)<br>*FGFR2* N549K<br>*PIK3CA* G1049R<br>*PTEN* K125N<br>*ARID1A* Q2115fs*33<br>*CHD4* R975H<br>*CTCF* S282fs*21<br>*MLL3* S2123*<br>*TP53* Y163C	Lenvatinib, Everolimus	No	・Lenvatinib is a potent FGFR inhibitor (**Supplemental Table S1**).<br>・*PIK3CA* alteration can be targetable with mTOR inhibitor (everolimus)	9
11	Gastroesophageal cancer	(FM, tissue)<br>*FGF19* amplification<br>*FGF3* amplification<br>*FGF4* amplification<br>*CCND1* amplification<br>*CDK6* amplification<br>*CDKN2A/B* loss<br>*PIK3CA* amplification<br>*SOX2* amplification<br>*PIK3CG* amplification<br>*PRKCI* amplification<br>*TERC* amplification<br>*TP53* G245D<br>TMB: intermediate (7Muts/Mb)	Lenvatinib, Palbociclib, Nivolumab	No	・Lenvatinib is a potent FGFR inhibitor (**Supplemental Table S1**).<br>・*CDK6* and *CCND1* amplification can be targeted with CDK4/6 inhibitor (palbociclib).<br>・Patients with intermediate TMB could benefit from anti-PD-L1 inhibitor (nivolumab).	
12	Gastroesophageal cancer	(FM, tissue)<br>*FGFR2* amplification<br>*TP53* A159V	Lenvatinib	No	Lenvatinib is a potent FGFR inhibitor (**Supplemental Table S1**).	
13	Ovarian cancer	(FM, tissue)<br>*FGFR4* amplification<br>*FLT4* amplification<br>*PDGFRB* amplification<br>*CDK6* amplification<br>*TP53* K132R<br><br>(GH, blood)<br>*TP53* K132, K120M<br>*PIK3CA* E545K<br>*MET* amplification<br>*PDGFRA* amplification<br>*KIT* amplification	Lenvatinib, Palbociclib	Yes	・Lenvatinib is a potent FGFR inhibitor (**Supplemental Table S1**).<br>・*CDK6* amplification may be targetable with CDK4/6 inhibitor (palbociclib).	
14	Gastrointestinal stromal tumor	(FM, tissue)<br>*KIT* K558_E562del, N822K, V654A<br>*ARID1A* truncation<br>*NOTCH2* P6fs*27<br>TMB: intermediate (7Muts/Mb)<br><br>(GH, blood)<br>*FGFR1* amplification<br>*MYC* amplification<br>*ERBB2* amplification	Lenvatinib, Pembrolizumab	Yes	・Lenvatinib is a potent FGFR inhibitor (**Supplemental Table S1**).<br>・Patients with intermediate TMB could benefit from PD-L1 inhibitor (pembrolizumab).	8
15	Adenoid cystic carcinoma	(FM, tissue)<br>*FGF19* amplification<br>*FGF3* amplification<br>*FGF4* amplification<br>*CCND1* amplification<br>*FANCA* F1263del<br><br>(GH, blood)<br>*FGFR1* amplification	Lenvatinib, Palbociclib	No	・Lenvatinib is a potent FGFR inhibitor (**Supplemental Table S1**).<br>・*CCND1* amplification may be targetable with CDK4/6 inhibitor (palbociclib).	
16	Undifferentiated sarcoma	(FM, tissue)<br>*FGFR3* amplification<br>*AKT2* amplification<br>*BRCA2* R1190W<br>*CCNE1* amplification<br>*CDK4* amplification<br>*MDM2* amplification<br>*AR* amplification	Pazopanib, Everolimus	No	・Pazopanib is a potent FGFR inhibitor (**Supplemental Table S1**).<br>・*AKT2* amplification may be targetable with mTOR inhibitor (everolimus).	10,11

Abbreviations: FM, Foundation Medicine; GH, Guardant Health; Muts, mutations; TMB, tumor mutation burden; PARP, Poly (ADP-ribose) polymerase; mTOR; mammalian target of rapamycin

References for Supplementary Table S2

1. Yang H, Liu Z, Wang Y, et al. Olaparib is effective for recurrent urothelial carcinoma with BRCA2 pathogenic germline mutation: first report on olaparib response in recurrent UC. *Ther Adv Med Oncol*. 2020;12:1758835920970845.

2. Garje R, Vaddepally RK, Zakharia Y. PARP Inhibitors in Prostate and Urothelial Cancers. *Front Oncol*. 2020;10:114.

3. Lau DK, Tay RY, Yeung YH, et al. Phase II study of everolimus (RAD001) monotherapy as first-line treatment in advanced biliary tract cancer with biomarker exploration: the RADiChol Study. *Br J Cancer*. 2018;118(7):966-971.

4. Wu CE, Chen MH, Yeh CN. mTOR Inhibitors in Advanced Biliary Tract Cancers. *Int J Mol Sci*. 2019;20(3):500.

5. Ahn ER, Mangat PK, Garrett-Mayer E, et al. Palbociclib in Patients With Non-Small-Cell Lung Cancer With CDKN2A Alterations: Results From the Targeted Agent and Profiling Utilization Registry Study. *JCO Precis Oncol*. 2020;4:757-766.

6. Min A, Kim YJ, Lee M, Lee KH, Im SA. Abstract PS5-40: CDKN2A loss can be a predictive marker of palbociclib in breast and gastric cancer. *Cancer Res*. 2021;81(4 Supplement):PS5-40.

7. Schettini F, De Santo I, Rea CG, et al. CDK 4/6 Inhibitors as Single Agent in Advanced Solid Tumors. *Front Oncol*. 2018;8:608.

8. Galuppini F, Dal Pozzo CA, Deckert J, Loupakis F, Fassan M, Baffa R. Tumor mutation burden: from comprehensive mutational screening to the clinic. *Cancer Cell Int*. 2019;19(1):209.

9. Barra F, Evangelisti G, Ferro Desideri L, et al. Investigational PI3K/AKT/mTOR inhibitors in development for endometrial cancer. *Expert Opin Investig Drugs*. 2019;28(2):131-142.

10. Tian T, Li X, Zhang J. mTOR Signaling in Cancer and mTOR Inhibitors in Solid Tumor Targeting Therapy. *Int J Mol Sci*. 2019;20(3):755.

11. Song M, Bode AM, Dong Z, Lee MH. AKT as a Therapeutic Target for Cancer. *Cancer Res*. 2019;79(6):1019-1031.

Supplementary Figure S1. STROBE diagram identifying eligible study patients from MSK-IMPACT Clinical Sequencing Cohort.

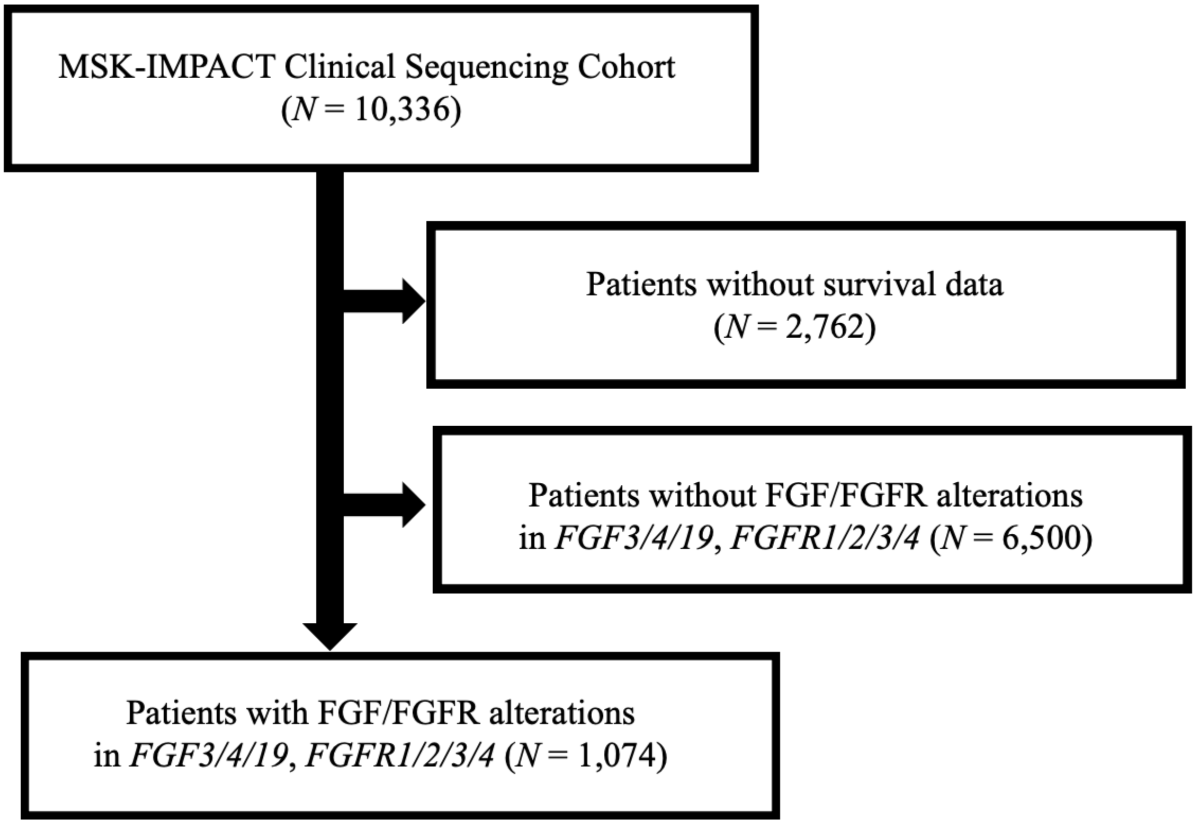

FGF6, *FGF23*, *FRS2* genes that were found in the UCSD cohort were not listed since they were not assessed in the MSK-IMPACT Clinical Sequencing Cohort.

Supplementary Figure S2. Patient flow of 858 cases discussed at UCSD Molecular Tumor Board (MTB).

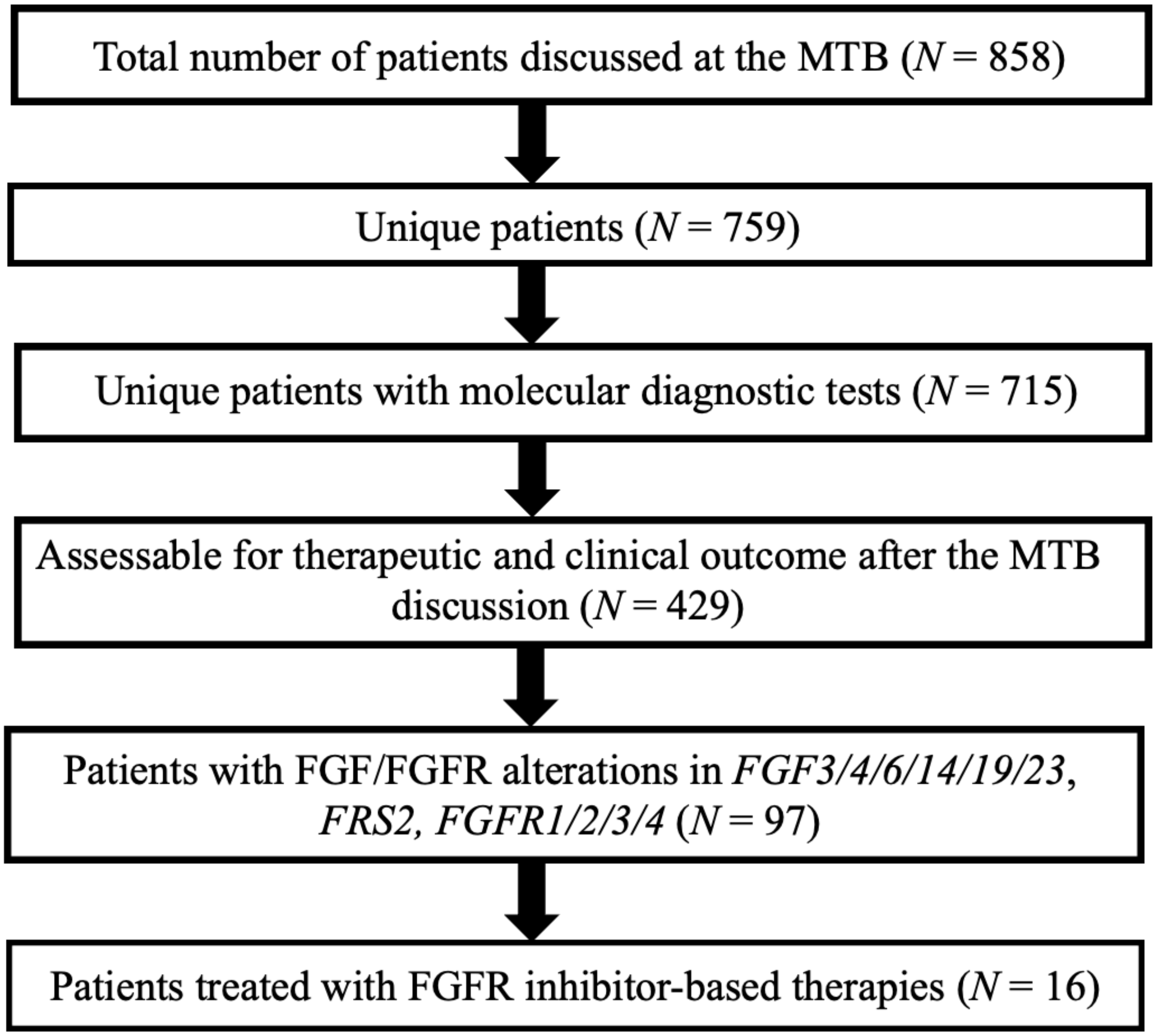

Supplement: Supplementary Table [file mmc1.pdf]
